# Estimating the Necessary Amount of Driving Data for Assessing Driving Behavior

**DOI:** 10.3390/s20092600

**Published:** 2020-05-02

**Authors:** Anna-Maria Stavrakaki, Dimitrios I. Tselentis, Emmanouil Barmpounakis, Eleni I. Vlahogianni, George Yannis

**Affiliations:** Zografou Campus, National Technical University of Athens, 5 Iroon Polytechniou Str, 157 73 Athens, Greece; dtsel@central.ntua.gr (D.I.T.); manosbar@central.ntua.gr (E.B.); elenivl@mail.ntua.gr (E.I.V.); geyannis@central.ntua.gr (G.Y.)

**Keywords:** driving data collection, driving behavior, driving assessment, smartphone data

## Abstract

The aim of this paper was to provide a methodological framework for estimating the amount of driving data that should be collected for each driver in order to acquire a clear picture regarding their driving behavior. We examined whether there is a specific discrete time point for each driver, in the form of total driving duration and/or the number of trips, beyond which the characteristics of driving behavior are stabilized over time. Various mathematical and statistical methods were employed to process the data collected and determine the time point at which behavior converges. Detailed data collected from smartphone sensors are used to test the proposed methodology. The driving metrics used in the analysis are the number of harsh acceleration and braking events, the duration of mobile usage while driving and the percentage of time driving over the speed limits. Convergence was tested in terms of both the magnitude and volatility of each metric for different trips and analysis is performed for several trip durations. Results indicated that there is no specific time point or number of trips after which driving behavior stabilizes for all drivers and/or all metrics examined. The driving behavior stabilization is mostly affected by the duration of the trips examined and the aggressiveness of the driver.

## 1. Introduction

Human factors such as driving under the influence of drugs or alcohol, distraction and inattention, speeding, aggressiveness and fatigue are proven to be the basic cause of road crashes, with a percentage of 65%–95% [1,2,3,4]. The rest of the factors that have an impact on crash probability include road environment (pavement, road signs, weather conditions, road design etc.), seatbelt use and vehicles (equipment and maintenance, damage etc.) as well as combinations of all three contributory factors [5,6].

Among the factors that relate to humans’ actions and reactions on the road, aggressiveness and distraction in driving behavior are of particular interest, as they become easier to monitor and study using the latest advances in technology [7,8,9]. More specifically, literature related to monitoring driving behavior using modern technology has centered to three attributes describing unsafe driving behavior, namely mobile phone usage, driving above the speed limit (speeding) and harsh driving [10,11]. Using the mobile phone while driving greatly influences driving behavior, as drivers show greater changes in speed, more fluctuations in the accelerator pedal position and they report a higher level of workload, regardless of the difficulty level of the conversation [10,12]. The same study proposed that drivers also tend to choose longer distances between vehicles and their reaction times are significantly increased. Driving above the speed limit is another significant factor that can lead to a crash (e.g., covering greater distance in case of a hazard, loss of control). According to [13], speeding is a contributing factor to 10% of total crashes and over 30% to fatal crashes. Finally, harsh events, namely acceleration, braking and turns, are important indicators for the assessment of driving risk, especially for assessing aggressiveness of driving [14]. These characteristics are strongly correlated with an unsafe distance from adjacent vehicles, accidental occurrences, lack of concentration, increased reaction time, poor driving judgment or low experience and participation in high risk situations. The association between harsh acceleration and harsh braking with dangerous driving has been highlighted in the scientific work published by [11,15,16,17] and has been widely recognized by the insurance and telecom industry [18].

The rapid technological progress, especially in Telematics [15,19], as well as the ever-increasing penetration and use of information technology by drivers (e.g., smartphones), can contribute to a deeper understanding and prevention of the factors that may lead to “near-misses” or actual road crashes through accurate monitoring, recording, analysis and assessment of driving behavior. Until recently, it was extremely difficult to collect and manage real-time data and, therefore, to study the relationship between driving behavior, travel behavior and the probability of crash involvement. This happened mostly due to the high cost of real-time driving data recording systems, data programs, cloud computing services, the inability to accumulate and exploit massive data bases (“Big Data”) for transport and traffic management purposes [20,21] and the low penetration rate of smartphones and social networks.

Nowadays, high quality real-time data can be collected in an efficient way in order to model both individual and total crash risk. With recent developments in tracking technology, new data collection methods, such as In-Vehicle Data Recorders (IVDRs) and smartphones, have emerged, giving the opportunity for large scale and real time monitoring and assessing of the actual driving behavior. In most studies, data are recorded by either On-Board-Diagnostics (OBD) [21] or smartphone devices [22] and transmitted to a central database for processing and analysis [23,24]. This allows for the development of special indicators to estimate driver’s travel and driving behavior. However, the exact size of the driving data that need to be collected and evaluated to determine the driving behavior with sufficient precision has not yet been determined. Both small and large data samples are likely to lead to questionable results by acquiring a sample either biased or computationally expensive to analyze, thus, making it important to investigate the amount of driving data that should be recorded by each participant in the experiment.

The aim of this paper was to develop a methodology for estimating the amount of driving data required to be collected for each driver to evaluate his driving behavior. Nowadays, data are of incalculable worth, as they can reveal and/or help us better understand driving behavior. Insufficient data can lead to misleading conclusions and biased results. On the other hand, collecting too much data, besides the increased experimental and computational costs, can be quite misleading [25]. Therefore, we examined whether there is a specific discrete time point beyond which the characteristics of driving behavior are stabilized over time and, as a result, a clear picture of driver’s behavior has been acquired. This amount was defined as the total driving duration and/or the number of trips that need to be recorded for each driver in order to obtain a clear picture regarding where the rate of driving characteristics (e.g., per kilometer or per minute) converges to a fixed point.

## 2. Materials and Methods

### 2.1. Data Collection

The basis of this framework is an innovative data collection system that continually records real-time driving behavior data of each participant using smartphone sensors. The driving behavior of 68 drivers was monitored and analyzed using several statistical tools to determine the minimum observation time for each driver and the potential to group drivers based on their driving aggressiveness.

Data were collected using the OSeven mobile application for both iOS and Android devices [26]. The application does not require any user engagement, and therefore, starts to collect raw smartphone sensor data from the built-in accelerometer, magnetometer, gyroscope and GPS during a trip. The accelerometer values are in m/s^2^ counting the gravity acceleration and the gyroscope values in rad/sec counting the angular velocity. Both sensors record data along three axes (x, y, z). Moreover, the specific app can automatically identify when the user has completed the trip and send the data to the servers of OSeven Telematics for processing through machine learning algorithms. Participants in the experiment should have a smartphone with built-in accelerometer, gyroscope and magnetometer sensor while commuting. For the specific work, data were collected with 1 Hz frequency. Users were very positive in using the app and participating in the experiment, since data were anonymized and no further user engagement while traveling was required.

The database used consisted of 21,610 separate trips collected from 68 drivers, which were chronologically ordered to observe the change in the magnitude of driving behavior characteristics over time. It should be noted that all the provided data was processed by OSeven Telematics, thus, no raw data processing was implemented in this study (i.e., converting data from the gyroscope and accelerometer to harsh braking events).

### 2.2. Main Risk Factors in Crash Research

In road crash literature [16], some of the travel and driving risk indicators that have been identified are: the total distance driven by the user, meaning that the higher the mileage, the higher the risk [15]., the road network type, as increased crash frequency can be observed in the cities, but increased crash severity can be observed in rural areas and highways. Furthermore, drivers are more likely to cause a crash during the so called “risky hours” or when they are driving in an unfamiliar environment (infrequent trips). Vehicle type and weather conditions are also considered as driving risk indicators, together with the seatbelt use and mobile phone use while driving. Lastly, the same study [16] indicated that harsh driving (e.g., harsh braking, acceleration or cornering) and speeding expressed either as a percentage of kilometers/time driving over the speed limit or a percentage of speeding are important indicators regarding travel and driving risk.

On a research level, there are several indicators both for travel behavior (vehicle maintenance condition, safety rating of the vehicle from the IIHS (Insurance Institute for Highway Safety)) and driving behavior (harsh cornering, alcohol, ecological driving etc.) that affect crash risk as well, but are not yet incorporated in risk modeling. Eco-driving for instance, is a significant factor for crash risk estimation [27]. According to the manufacturer’s specifications, conclusions can be drawn about how a person is driving (aggressively, over the speed limits etc.) if fuel consumption estimated by the manufacturer is compared to the real fuel consumption recorded. Furthermore, the simultaneous existence of two driving traits, namely, excessive speeding during the risky hours timeframe or braking harshly while using the mobile phone, might excessively affect crash risk. It should be mentioned, however, that some of the indicators mentioned above, such as the use of alcohol, cannot be considered in the driving behavior models of the present analysis as they cannot be captured efficiently yet. Nevertheless, it is very likely for scientists to be able to monitor these factors in an easy and reliable manner in the near future and therefore exploit this information as well.

As for the indicators used in today’s Usage Based Insurance (UBI) models, the predominant among them are mileage, speeding, road network type and risky/rush hours driving. It is anticipated that apart from these, more behavioral parameters e.g., the number of sudden braking/acceleration/cornering events, mobile phone usage etc., will be used a lot in future models because they represent the crash probability better.

It can be deduced from the above that the most significant human factors that were found to affect driving risk, which will be further used to identify the amount of data that should be collected to understand a driver’s behavior, are: i. mobile phone distraction, ii. speed limit exceedance and iii. the number of harsh braking and acceleration events that occurred while driving [16]. Harsh cornering is not explicitly utilized as a metric in this study, as its contribution as a driving behavior indicator can be grouped in that of the harsh braking events.

### 2.3. Methodological Approach

#### Identifying Driving Behavior Convergence

As previously mentioned, the driving metrics used in this study to identify driving behavior convergence are the number of harsh acceleration (HA) and braking events (HB), the time of mobile usage (MU) and the time of driving above the speed limit (SP). Cumulative sums of those metrics (per kilometer for harsh events and as percentage of driving duration for mobile usage and speeding) were used to reveal when driving characteristics stabilize or fluctuate around a fixed value over time. This trend is also captured in the convergence figures provided below.

The analysis was conducted on a trip basis, and three distinct trip duration categories were used (5-, 10- and 20-min trips). The variability of the above metrics is then examined to observe driving behavior evolution over time. For this purpose, the measures of simple moving average and volatility were used along with statistical limits (hart charts, [28]) and conditions that need to be met to identify convergence. We utilized the basic convergence principles of Shewhart charts, which set the “confidence intervals” for identifying the area where convergence was achieved.

For each of the sub-databases originating from the initial database of the 68 drivers, it was checked whether and when all of the following conditions were met simultaneously:

- The moving average is within the range “mean ± 1 * standard deviation.”
- For five consecutive trips, the percent change (in absolute terms) between successive values of the moving average is less than or equal to 1.5%.
- The value of the moving average in the corresponding trip is a local extreme (this criterion ensures that the neighboring values of the moving average are smaller or larger than the selected one, and therefore, it does not belong to a sequence of points that have a particular trend e.g., ascending or descending).

These criteria were separately applied on the cumulative sum measures and to their volatility measures. For each driver, each time step was iteratively checked to examine when the above criteria were met. The first trip, for which all of the above conditions are met, was assigned to the drivers’ database as the first time point at which the particular attribute converged to a certain value. At the same time, the values at which the cumulative sum metrics and their volatility converged were also recorded.

To calculate volatility, the ratio of the Gain/Loss of each driver was calculated and defined as the gain (= improvement) or the loss corresponding to drivers driving behavior among successive journeys. If k is the metric that is examined (number of HA events, duration of MU, duration of SP etc.), i is each driver (i=1,2,3,….,N) and t the number of his trips t={1,2,3,….,n}, then the Gain/Loss ratio for each trip is calculated as:

(1) $$r_{t,i}=ln\left(\frac{k_{t,i}}{k_{t-1,i}}\right)$$

This ratio is negative when a driver is improving their driving behavior (for example when the number of HA events per km is reduced compared to the previous trip and positive when the opposite happens).

Then, the magnitude of volatility is calculated as the standard deviation of the Gain/Loss ratio in order to examine how consistent the driver is between different trips introduced in [1] as follows:

(2) $$Volatility=\sqrt{\frac{\sum_{t=1}^{n}{\left(r_{t,i}-\bar{r_{i}}\right)}^{2}}{n-1}}$$

where $r_{t,i}$ is the Gain/Loss ratio for every trip *t* of every driver *i*, $\bar{r}$ is the mean value of the ratio Gain/Loss for the driver *i* and *n* is the number of his trips. In order to calculate the mean value of the ratio, the number of trips should first be defined. For example, if all trips of a driver are to be used, then volatility will be calculated compared to the whole sample. However, since the actual case is that different drivers had a different number of trips, using the whole sample would not be realistic. As a result, a constant moving window of 20 trips was chosen, taking into account that no driver and no characteristic can converge earlier than 20 trips. This is supported by the analysis of the data that also proved that none of the participants in the experiment exhibited a driving behavior that would allow them to converge earlier than 20 trips. Intuitively, this amount of data is the equivalent of monitoring an average driver for at least 2 weeks, which can be considered enough for statistical analysis. It is seen, therefore, that each value of volatility of driver *i* compared the driving behavior of each trip $r_{t,i}$ to the mean value $\bar{r_{i}}$ of the 19 next observed trips of the same driver.

Finally, the Shewhart control chart principles were used [28], which examine whether a variable remains stable over time and within two given upper and lower limits. The two limits, the upper control limit (UCL) and lower control limit (LCL), are defined as follows:

(3) UCL=Accepted value+k∗process standard deviation

(4) LCL=Accepted value − k∗process standard deviation

## 3. Results

The procedure described above is applied to the initial database of 68 drivers, for trips with an average duration of 5, 10 and 20 min. The analysis was conducted only for the above average trip durations, since the number of trips with a duration over 25 min is significantly lower, resulting to an extremely low number of trips for all drivers (less than three trips for 93% of the drivers). Therefore, no duration category above 20 min was used in the analysis since this would then lead to statistically insignificant and uncertain results. The final analysis performed included data from 29 drivers who were used to obtain the results illustrated in Table 1. These 29 drivers were those having sufficient number of trips in all trip duration categories examined in this study (5-, 10- and 20-min duration). The threshold used to determine whether or not an adequate number of trips has been recorded for a driver was 20 trips, which is equal to the moving window’s number of trips.

Table 1 presents the descriptive statistics of the minimum number of trips for all three trip duration categories studied. After applying the methodology, the specific number of trips after which convergence of the driving behavior metrics was established for the above 29 drivers. “Trip duration 5” refers to the trips that lasted less than 5 min, “Trip duration 10” to the trips that lasted between 5 and 15 min (average duration of sub-database: 10 min) and “Trip duration 20” to trips that lasted between 15 and 25 min (average duration of sub-database: 20 min). The results of Table 1 are grouped by trip duration category and drivers’ aggressiveness level i.e., the number of harsh acceleration/braking events per 100 km driven and the percentage of mobile usage and time speeding while driving.

The results in Table 1 demonstrate that no single time point at which driving behavior stabilizes exists for all drivers and/or all driving behavior metrics. This finding, although expected, because drivers differ in driving aggressiveness, hints that the identification of a driver’s observation time—before forming his driving profile—should be preceded by an analysis of the aggressiveness profile. Results indicate that the most aggressive drivers (i.e., the ones with a larger number of harsh events per km) tend to converge at a faster rate than the less aggressive drivers, confirming the results of the literature [9,15]. More specifically, it is noticed that, on average, more aggressive drivers tend to converge (for all metrics and their volatility) at around 80 trips, while less aggressive drivers converge at around 100 trips. For instance, the average number of trips required that the convergence of all metrics of trips with an average duration of 5 min is 102 and 86 for less and more aggressive drivers, respectively. Consequently, the metrics that generally converge with the slowest rate refer to cautious drivers and are usually the volatility of HA events, the number of HB events per kilometer and the percentage of MU while driving. In addition, slower convergence rate in their volatility measures as well (regarding all metrics) was observed for cautious drivers.

**Figure 1:**
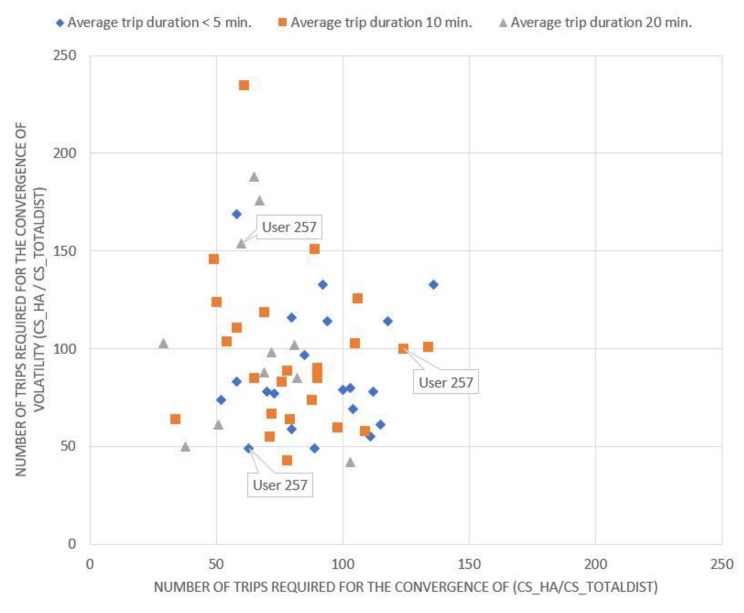
Minimum number of trips required for the number of harsh acceleration events per km rate to converge.

Apart from the aggressiveness, the number of trips for which drivers are required to be monitored to extract their overall driving profile also varied in terms of the average duration of the trips being studied. For example, it is clear from Figure 1 that the minimum number of trips required for convergence is generally smaller for trips with an average duration of 20 min than the corresponding number for shorter trips (e.g., 5- or 10-min trips). This means that, even for the same driver, the rate of convergence of the same characteristic varied considerably, depending on the average duration of the trips that are being studied, e.g., 10 min or 20 min. Driver with ID “257” is highlighted in Figure 1 for the three different trip durations mentioned above. Thus, it becomes apparent that the relative position of the same driver on the chart might be altered even for the same characteristic, and thus, it can be said that the minimum number of trips that should be collected for each driver depends not only on their aggressiveness, but also on the duration of their trips.

The driving behavior metric that converges later for each driver is the critical driving characteristic that determines the minimum number of trips that need to be collected to obtain a clear picture for his driving behavior. In many cases, this may correspond to the magnitude of volatility of a characteristic, since the convergence rate of a characteristic for the same driver generally differs from the convergence rate of the volatility of the same characteristic Figure 1 illustrates the number of trips required for the convergence of the magnitude of the cumulative number of HA events to the cumulative total distance travelled versus the number of trips required for the convergence of the volatility measure of this magnitude for the three different duration categories studied.

**Figure 2:**
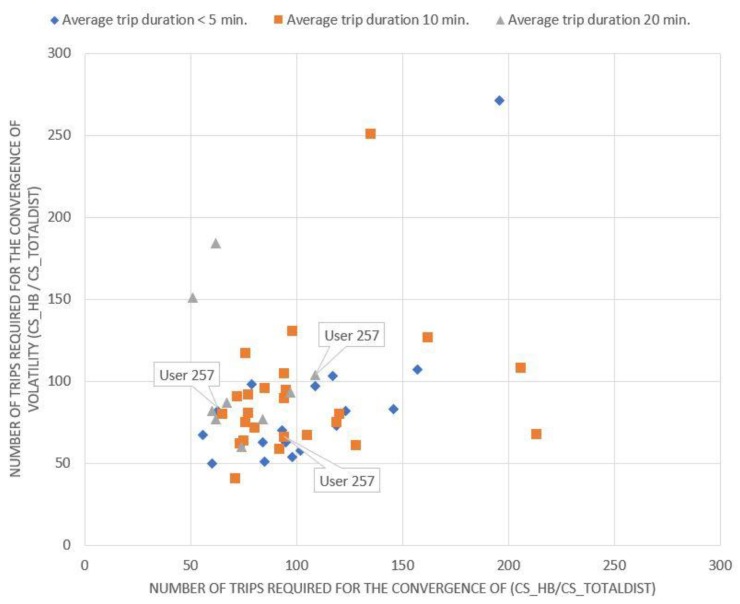
Minimum number of trips required for the number of harsh braking events per km rate to converge.

**Figure 3:**
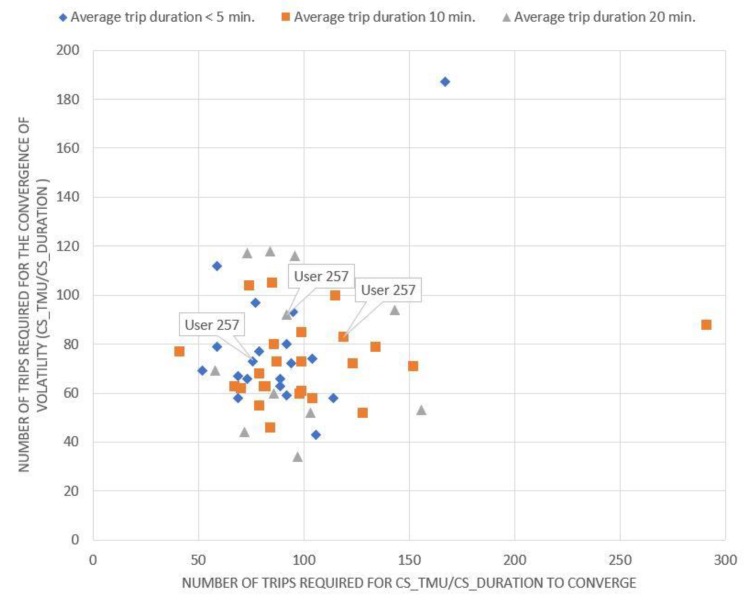
Minimum number of trips required for the percentage of time mobile usage rate to converge.

**Figure 4:**
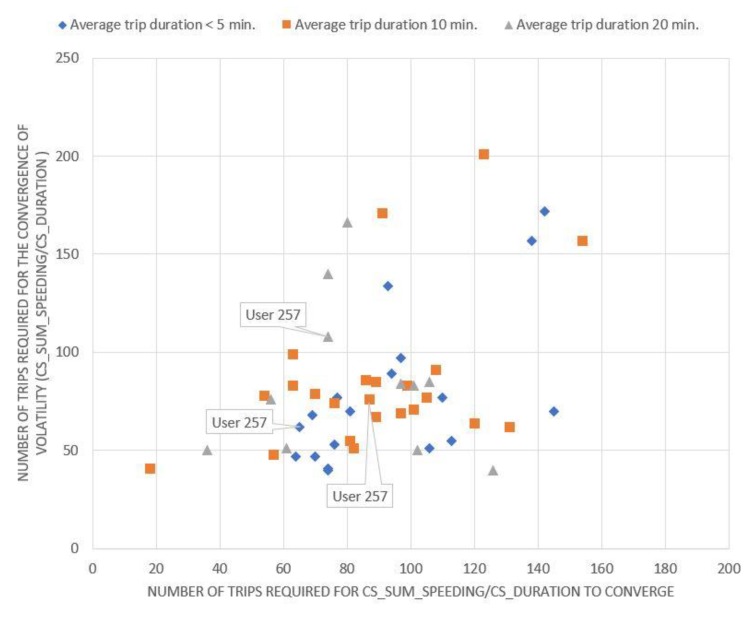
Minimum number of trips required for the percentage of time speeding rate to converge.

Evidently, if a driver is on the diagonal, the convergence rate of the number of HA events per kilometer (x-axis) is equal to the convergence rate of the volatility of the same magnitude (y-axis). If a driver is below the diagonal, the minimum number of trips that need to be collected depends on the number of HA events per kilometer, while if a driver is above the diagonal, it depends on the volatility of the same metric. Equivalent conclusions also arise from the examination of the corresponding charts (Figure 2, Figure 3 and Figure 4) for the other driving behavior metrics studied, namely the number of HB events, the percentage of time of mobile usage and the percentage of time speeding while driving.

As indicated from Figure 1, Figure 2, Figure 3 and Figure 4, if a driver needs to be monitored for more than 120 trips until the volatility measure of a driving metric converges, the driving behavior is considered to have converged relatively slowly. On the other hand, if the volatility measure converges in less than 60 trips, the driving behavior is considered to have converged relatively fast. However, several differences can also be observed between the above-mentioned figures. It is obvious that for Figure 3; Figure 4, drivers are more concentrated around a specific area, with only a few of them being dispersed. This means that most drivers appear to have a converging behavior at roughly 50–120 trips for mobile usage and speeding, which is considered to be a relatively large range. Therefore, it can be inferred that there is no specific time point or number of trips at which driving behavior metrics converge to an average value.

On the contrary, the drivers described in Figure 2 appear to be more dispersed with no specific pattern in the cloud of points formed. This indicates that their behavior and volatility towards HB events varies between different duration categories, differs from those noticed in Figure 3 and Figure 4 and is more similar to that noticed in Figure 1. These results are also confirmed by Table 1.

Table 2 summarizes the results of the analysis performed on the convergence rates of the four driving metrics examined, which are categorized as fast or slow based on the minimum number of trips required to be collected. It also illustrates the aggressiveness and volatility limits noticed in each convergence rate group. To estimate the numbers of Table 2 the minimum and maximum values of Table 1 were taken into consideration, as well as the median and the standard deviation. These values were rounded to provide a characterization of drivers as aggressive/cautious and stable/volatile. The 42.24% of the drivers were found to have fast convergence rate regarding their volatility measure, while for the driving metrics this magnitude ranges from 13.79% (for HB events per kilometer) to 24.14% (for HA events per kilometer and percentage of time speeding). Regarding slow convergence, both for the volatility measure and the driving behavior metrics the percentage of drivers in this category ranges from just above 20% to 27.59%, except from the metric of HA events per kilometer, where the corresponding percentage is 10.34%. Table 2 also indicates that over 35% of the drivers were found to have a stable driving behavior in general, and over 30% of them were also cautious regarding HA events per kilometer and mobile usage. The highest percentages of aggressiveness, though, were found in the above-mentioned characteristics, being 17.24% and 21.84% of the drivers accordingly.

**Figure 5:**
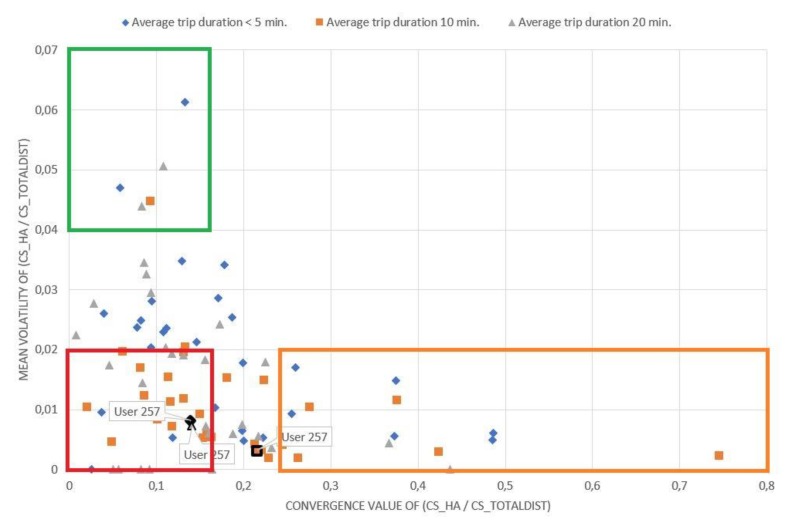
Aggressiveness versus volatility of driving behavior—harsh acceleration events.

The aggressiveness and volatility of drivers were determined from the average values at which driving behavior characteristics and their volatility converge. A driver may be cautious regarding the matric being studied (i.e., the mean value at which this characteristic converges is small, for example the number of HB events per kilometer is less than 0.01), but at the same time exhibiting significant variations/fluctuations in the travel-related behavior (high volatility index, for example the volatility of HB events per kilometer is greater than 0.05), and vice versa. This is made clear in Figure 5, which presents HA events per kilometer, in combination with Table 2.

Figure 5 presents the mean volatility measure (y-axis) in relation to the convergence value (mean) of the cumulative number of HA events per kilometer (x-axis) to which each driver converges for the three different trip duration categories considered. As can be seen in Table 2 and regarding the number of HA events, drivers in the area of Figure 5 with a mean volatility and metric convergence value of less than 0.02 and 0.17, respectively, present a generally stable behavior (with few fluctuations) and a low number of harsh acceleration events. This is the area where non-volatile and cautious drivers belong regarding this driving characteristic. Accordingly, drivers in the area of average volatility and metric value of more than 0.05 and less than 0.17, respectively, were characterized as volatile, cautious drivers. On the other hand, those drivers in the area with an average volatility and metric value of less than 0.02 and greater than 0.23, respectively, were characterized as non-volatile, aggressive drivers. It is noted that all the above observations refer to the specific driving characteristic of HA events.

Figure 1 shows that the aggressiveness and volatility of a driver depend to some extent on the average duration of the trips being studied. Even for the same driver, there are differences depending on trip duration, e.g., 10 min or 20 min. Nonetheless, these differences are usually non-significant, i.e., drivers seem to maintain approximately the same behavior and behavioral volatility in terms of a driving characteristic, regardless of the average duration of the trips being studied.

The driver with username “257” has been highlighted as well in Figure 5, for the three different trip durations studied in this paper, illustrating whether the same driver changes his relative position on the chart. Equivalent conclusions also arise from the examination of the corresponding figures for the other driving metrics studied, namely the HB events, the percentage of time of mobile usage and the percentage of time speeding while driving.

Investigating the critical driving characteristic (i.e., the one that converges more slowly than the rest) for determining the required amount of driving data to be collected for each driver, out of the 29 that were finally used in the analysis resulted in Table 3. This table shows that for the majority of drivers the critical characteristic is the volatility of the number of HA events per km as well as the percentage of time of mobile usage while driving. The number of HB events per km and its volatility follow, while for a few drivers, it seems that the percentage of time speeding and its volatility is the critical characteristic.

**Figure 6:**
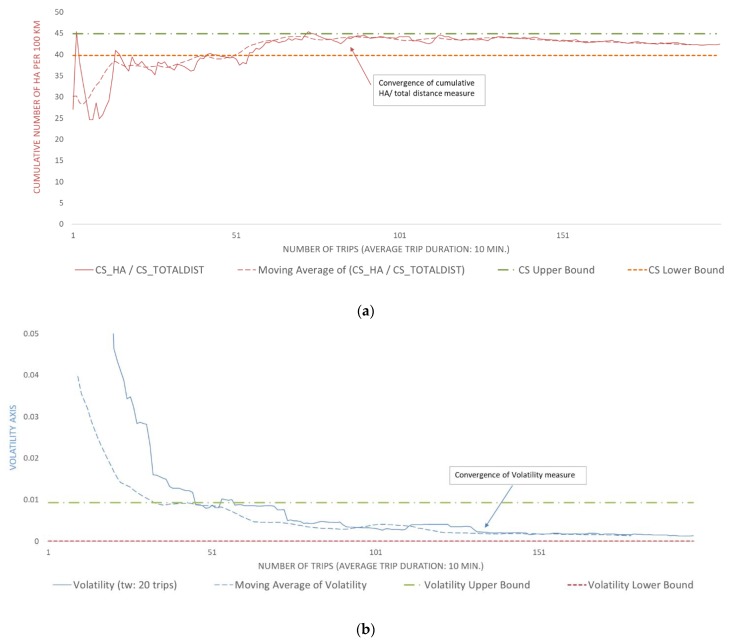
(**a**) Convergence plot of the cumulative harsh acceleration events per km for user “9.” (**b**) convergence plot of the volatility of harsh acceleration events rate for user “9.”

**Figure 7:**
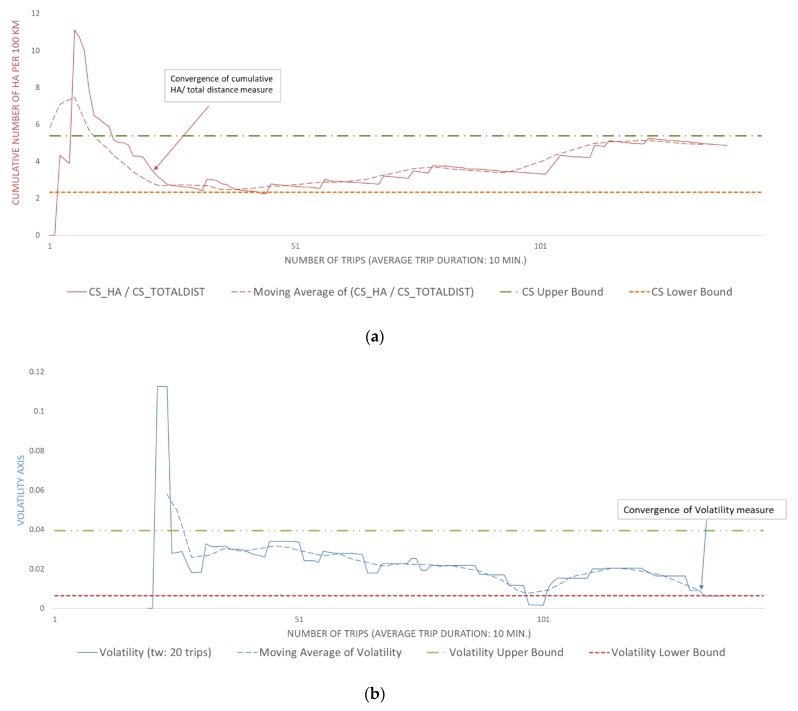
(**a**) Convergence plot of the cumulative harsh acceleration events per km for user “154.” (**b**) Convergence plot of the volatility of harsh acceleration events rate for user “154.”

Figure 6 and Figure 7 are indicatively provided to illustrate and compare the convergence of two drivers regarding the number of harsh acceleration events, for an average trip duration of 10 min. These two drivers, users “9” and “154,” were randomly selected from the driving sample of the more and less aggressive drivers, respectively, using a random number generator to produce random user IDs. The temporal change in driving characteristics and their volatility as well as the time points at which driving behavior is converged can be noticed in both figures. The results indicate that the HA events rate of user “9” converged after the 76th trip and that the volatility of the same metric converged after the 132nd trip. As for user “154,” the methodology indicated that convergence for the HA events rate occurred after the 134th trip and after the 22nd trip for the volatility of the same metric.

## 4. Discussion and Conclusions

This work attempted to identify a discrete time point or number of trips after which additional driving data do not add a significant insight for driver’s general behavior. To this end, a methodology was developed and applied on detailed data collected from smartphone sensors. Various mathematical and statistical tools were used to process the data and determine the time point at which behavior converges. Initially, the cumulative sum of the number of HA/HB events per kilometer, the percentage of time of mobile usage while driving and the percentage of time speeding was created. This procedure was followed by the calculation of the driver’s behavioral volatility of the above-mentioned metrics and the use of moving averages of those metrics to determine convergence and the number of trips required for each metric to converge. Data analysis indicated that for a certain driving characteristic, the amount of time required to be collected largely depends on the aggressiveness and stability of the overall driver’s behavior, as well as the average duration of the trips being studied.

In particular, more aggressive drivers require less monitoring than cautious drivers do. It is inferred that further investigation of the aggression level of drivers and the driving environment should be preceded. Aggressive drivers are those with a high number of harsh events and high percentages of time driving over the speed limit. The analysis revealed that drivers with high average convergence values of acceleration events per kilometer also show high average convergence values of HB events per kilometer, while those with low average convergence values of acceleration events per kilometer also exhibit low average convergence values of braking events per kilometer.

Apart from aggressiveness, another driving characteristic that influences the time of convergence is the stability or volatility of driving behavior. Knowledge of drivers’ behavioral volatility is of paramount importance when studying driving behavior, as it provides important insights into their overall experience and the difference in behavior between trips. The investigation of the critical observation metric of each driver (i.e., the one that converges slower than the rest) showed that in the majority of cases, the volatility of behavior was the most critical parameter, reaching a level of 44.44% for HA events per kilometer in trips with average duration of 5 min.

Regarding a specific driving characteristic, it is extremely difficult to identify the exact time point where a driver’s behavior converges when the trips being studied do not have a similar duration. Since data collected are related to driving behavior characteristics on a trip level, overall behavioral change could not be analyzed and investigated using time series analysis methods, as the driving duration between successive journeys may vary significantly. It is therefore necessary to group trips travelled by driving duration and sort them in chronological order. The duration of the trips analyzed was also found to affect the point of convergence of a driver’s behavior. In particular, it was shown that the same driver may exhibit significant differences in the amount of data required to be collected with respect to a particular driving characteristic when considering journeys of different average driving duration.

On the other hand, the duration of the trips analyzed does not significantly affect the average metric value at which a driver’s behavior converges for a specific driving characteristic. In particular, when considering the driving behavior of a specific driver with respect to a particular driving characteristic, no significant changes are noticed in the value at which this driving characteristic converges at, as the average trip duration changes. Consequently, a different monitoring period is required when short or slightly longer trips are being studied despite the fact that drivers might present similar behavior. Nevertheless, this may be due to the fact that drivers who participated in the experiment conducted mostly drove on urban networks and, therefore, the road environment and driving conditions were familiar to them. It should be noted that although the trip duration was studied, no safe conclusion can be drawn regarding the relationship between the type of road network and the amount of data that should be collected for driving behavior analysis, as this is something that should be examined separately.

Some of the limitations of this study that should be addressed include the inability to record other significant crash risk factors such as alcohol and drug use. Moreover, a significant number of drivers were eliminated from this study and trip duration over 20 min was not studied because of data limitations coming from the sample size. Therefore, a larger sample of drivers is suggested to be exploited in the future in order to overcome such limitations.

The findings presented in this work could be exploited either to provide feedback to drivers on how to improve their driving behavior or to improve the services provided by insurance companies and car industries, which would bring multiple and significant benefits to the society. Additionally, they could be used further by researchers to efficiently design their experiment, especially when it comes to collecting naturalistic driving behavior data.

## Figures and Tables

**Table 1:** Aggregated table of minimum number of trips required for convergence.

Trip Duration	Metric Limits	Metric					Volatility					No of Drivers
		min	max	Average	Median	StDev	min	max	Average	Median	StDev	
**5**	**HA ≤ 15**	63	112	92	92	17	49	169	95	81	35	27
	**HA > 15**	52	136	86	85	27	36	97	65	70	19	
	**HB ≤ 5**	60	196	110	109	44	50	271	85	70	58	
	**HB > 5**	56	157	97	94	31	52	103	81	85	19	
	**MU ≤ 10%**	76	167	102	94	26	43	112	76	75	18	
	**MU > 10%**	52	104	76	73	17	38	187	73	67	38	
	**SP ≤ 3.5 %**	69	145	104	104	29	41	157	79	70	34	
	**SP > 3.5 %**	64	138	86	76	23	34	172	65	50	38	
**10**	**HA ≤ 15**	58	109	84	84	14	74	235	115	103	40	29
	**HA > 15**	49	134	80	75	26	43	119	67	62	22	
	**HB ≤ 6**	71	213	118	97	50	62	251	102	90	47	
	**HB > 6**	65	135	90	77	22	41	96	69	66	18	
	**MU ≤ 7%**	41	291	110	98	61	58	203	86	79	35	
	**MU > 7%**	67	134	95	87	21	46	105	64	63	16	
	**SP ≤ 5 %**	18	154	89	88	32	62	201	99	83	46	
	**SP > 5 %**	53	123	85	85	23	41	99	68	71	19	
**20**	**HA ≤ 12**	14	103	61	69	35	61	188	117	102	44	16
	**HA > 12**	29	81	59	63	17	42	50	46	46	6	
	**HB ≤ 5**	84	102	94	97	9	60	184	102	87	40	
	**HB > 5**	51	109	69	65	17	-	-	-	-	-	
	**MU ≤ 10%**	72	156	106	96	31	34	118	73	65	30	
	**MU > 10%**	58	103	80	80	19	38	116	65	41	44	
	**SP ≤ 10 %**	56	126	87	88	27	40	166	85	83	40	
	**SP > 10 %**	36	106	71	74	26	46	52	49	49	4	

**Table 2:** Aggressiveness, volatility limits and convergence rate of driving behavior.

	Minimum Required Number of Trips		Average Conversion Rate of Driving Characteristics and Volatility			
	Fast Convergence	Slow Convergence	Cautious	Aggressive	Stable	Volatile
**Harsh Acceleration events per km**	<50 (24.14%)	>120 (10.34%)	<0.11 (33.33%)	>0.23 (17.24%)	-	-
**Harsh Braking events per km**	<60 (13.79%)	>140 (20.69%)	<0.01 (5.75%)	>0.12 (9.20%)	-	-
**Percentage (%) of Time Mobile Usage**	<50 (17.24%)	>120 (27.59%)	<0.04 (32.18%)	>0.16 (21.84%)	-	-
**Percentage (%) of time Speeding**	<50 (24.14%)	>120 (24.14%)	<0.02 (12.64%)	>0.14 (9.20%)	-	-
**Volatility**	<60 (42.24%)	>120 (21.55%)	-	-	<0.005 (35.63%)	>0.05 (23.75%)

**Table 3:** Cumulative table of percentages of drivers and their critical characteristic for each duration.

Critical Characteristic								
	Harsh Acceleration Events per km		Harsh Braking Events per km		Percentage (%) of Time Mobile Usage		Percentage (%) of Time Speeding	
Average Trip Duration	Cumulative Sum	Volatility	Cumulative Sum	Volatility	Cumulative Sum	Volatility	Cumulative Sum	Volatility
**5 min**	29.63%	44.44%	29.63%	29.63%	25.93%	14.81%	14.81%	11.11%
**10 min**	24.14%	27.59%	20.69%	41.38%	37.93%	17.24%	17.24%	13.79%
**20 min**	18.75%	37.50%	12.50%	18.75%	37.50%	18.75%	31.25%	25.00%
